# The central role of stem cells in determining plant longevity variation

**DOI:** 10.1016/j.xplc.2023.100566

**Published:** 2023-02-24

**Authors:** Omid Karami, Bernd Mueller-Roeber, Arezoo Rahimi

**Affiliations:** 1 Plant Developmental Genetics, Institute of Biology Leiden, Leiden University, Sylviusweg 72, 2333 BE Leiden, the Netherlands; 2 University of Potsdam, Institute of Biochemistry and Biology, Karl-Liebknecht-Straße 24–25, Haus 20, 14476 Potsdam, Germany

**Keywords:** stem cells, plant longevity, annual plants, perennial plants, axillary meristems

## Abstract

Vascular plants display a huge variety of longevity patterns, from a few weeks for several annual species up to thousands of years for some perennial species. Understanding how longevity variation is structured has long been considered a fundamental aspect of the life sciences in view of evolution, species distribution, and adaptation to diverse environments. Unlike animals, whose organs are typically formed during embryogenesis, vascular plants manage to extend their life by continuously producing new tissues and organs in apical and lateral directions via proliferation of stem cells located within specialized tissues called meristems. Stem cells are the main source of plant longevity. Variation in plant longevity is highly dependent on the activity and fate identity of stem cells. Multiple developmental factors determine how stem cells contribute to variation in plant longevity. In this review, we provide an overview of the genetic mechanisms, hormonal signaling, and environmental factors involved in controlling plant longevity through long-term maintenance of stem cell fate identity.

## Teaser

Longevity varies greatly among vascular plants, and this variation is highly dependent on the activity and fate identity of self-renewing stem cells within meristematic tissues. Despite considerable interest, there is no good understanding of the molecular mechanisms involved in the activity and fate of stem cells that contribute to variation in plant longevity.

## Introduction

Longevity in vascular plants, as in any other multicellular organism, is defined as the period during which plants remain viable. Plants display a huge variety of longevity patterns, ranging from a few weeks in several annual species up to thousands of years in some perennial species (Munné-Bosch, 2007, 2014). How longevity variation is structured in plants is a fundamental question with importance for evolution, species distribution, and adaptation to different environments (Lundgren and Des Marais, 2020). Although we can assume that distinct developmental and physiological characteristics underlie longevity, how plants control longevity at the molecular level is poorly understood.

Vascular plants are divided into two main groups: seedless and seed plants. The life cycle of both groups alternates between two generations: the haploid gametophyte generation and the diploid sporophyte generation (Bowman et al., 2016). In seedless vascular plants such as ferns, gametes (sperm and egg cells) are produced in the transient haploid gametophyte generation, whereas spores are produced via meiosis in the relatively long diploid sporophyte generation (Bowman et al., 2016). In seed plants, the sporophyte is the main plant body, and the gametophyte is typically much shorter-lived and dependent on the sporophyte (Frank and Scanlon, 2015; Gaillochet and Lohmann, 2015). By contrast, in seedless vascular plants, the gametophyte and sporophyte represent independent generations (Watkins and Cardelús, 2012).

Seed plants have evolved two main, opposing growth habits related to reproductive rate and survival probability. (i) Some plants reproduce only once in their lifetime, and they senesce at the whole-plant level and die, even under appropriate growth conditions. This growth habit is referred to as monocarpy. All annual plants but few perennial plants are monocarpic. Monocarpic perennials survive for several years but die after producing offspring once. (ii) Other plants do not die after a reproductive period; they typically produce multiple successful offspring over successive seasons during their lifetime and are referred to as polycarpic plants (Amasino, 2009). Polycarpic plants are usually perennial, entering the reproductive phase multiple times (Munné-Bosch, 2008; Thomas, 2013). Polycarpic perennial plants are divided into two main groups: non-woody (herbs) and woody perennials (trees and shrubs); each group shows remarkable diversity in morphology and mortality pattern (Jones et al., 2014).

Many monocarpic plants, such as petunia and tomato, tend to display polycarpic behavior when grown in tropical conditions, but they are typically grown as monocarpic plants because they cannot survive freezing temperatures during the winter (Friedman, 2020). Other monocarpic plants switch to polycarpy because of delays in seed germination, changes in relative growth rate, loss of apical dominance, thriving in harsh environments, or injury at the flowering and fruiting stages (Sosnová and Klimešová, 2009; Klimešová et al., 2020).

In many seed plant species, growth habit has switched from mono- to polycarpy, and vice versa, throughout evolution (Partridge and Harvey, 1988; Karl and Koch, 2013; Friedman, 2020), and this is considered the most frequent transition of growth habit in angiosperms (Amasino, 2009). The co-existence of mono- and polycarpic species in many genera suggests that the transition between polycarpic and monocarpic growth habits requires only small genetic (or epigenetic) changes. However, despite considerable interest in understanding the molecular mechanisms that control the switch between mono- and polycarpy, only a few genes associated with offspring-linked mortality in monocarpic plants and survival of polycarpic plants over many offspring productions have been identified to date.

Unlike animals, in which most organs are typically formed during embryogenesis, vascular plants can extend their life continuously, for many years in some species, producing new tissues and organs in apical and lateral directions via proliferation of stem cells located in specialized tissues called meristems (Gaillochet and Lohmann, 2015). Plant stem cells make an important contribution to longevity. Variation in plant longevity is strongly dependent on persistence of stem cells and maintenance of the fate of daughter cells that differentiate while forming organs (Munné-Bosch, 2007). Although it is well known how plants maintain populations of stem cells (Dodsworth, 2009; Bustamante et al., 2016; Somssich et al., 2016; Xue et al., 2020), less is known about the factors that control stem cell maintenance in the context of plant longevity variation. Understanding the molecular genetic mechanisms that underlie long-term maintenance of stem cell identity is fundamental to addressing basic variation in plant longevity. In this review, we provide an overview of the genetic mechanisms, hormonal signaling, and environmental factors involved in controlling plant longevity with respect to persistent pluripotency and maintenance of fate identity in stem cells.

## The constant activity of stem cells has a key role in plant longevity

Stem cells are self-renewing, undifferentiated cells capable of differentiating into various tissues and organs. Therefore, maintenance of constantly dividing stem cells is necessary for continued plant growth (Munné-Bosch, 2007). In seed plants, stem cells comprise a population of cells formed in diploid sporophytes and are lost in the haploid gametophyte. By contrast, stem cells in non-seed plants are typically single cells that develop in the gametophyte generation and are retained in the sporophyte (Imaichi, 2013). Different types of gametophyte stem cells persist in an undifferentiated state, self-renew through continuous cell division, produce daughter cells with the potential to differentiate into photosynthetic cells or cells forming sexual organs, and are eventually terminated in response to environmental signals or developmental cues (Imaichi, 2013; Conway and Di Stilio, 2020). Compared with the well-characterized regulatory mechanisms of stem cell maintenance in seed plants, the mechanisms underlying stem cell maintenance in non-seed plants are just beginning to be understood.

In seed plants, almost all postembryonic production of tissues and organs results from proliferation and differentiation of stem cells within meristematic tissues in the shoot apical meristem (SAM) (Uchida and Torii, 2019), root apical meristem (RAM) (Uchida and Torii, 2019), and vascular cambium meristem (VCM) (Tonn and Greb, 2017). In contrast to intensive research on stem cell maintenance in annual plants, the mechanisms that regulate stem cell maintenance and the constant activity of stem cells in perennial plants have received little attention. Answers to the question of how perennial plants can survive for many years could be obtained by understanding the molecular and cellular mechanisms that regulate constant stem cell activity in meristems of perennial plants.

### Role of the SAM

In seed plants, the SAM is responsible for continuously producing aboveground organs, including leaves, stems, and flowers (Gaillochet and Lohmann, 2015). After asymmetric cell division of a stem cell in the SAM, one of the daughter cells maintains stem cell identity and continues to be a stem cell (self-renewal), whereas the other daughter cell differentiates into different cell types to produce lateral organs such as leaves and flowers. The proliferative activity of stem cells and the fate of daughter cells typically depend on the interaction of stem cells with their immediate cellular microenvironments, which encompass the so-called stem cell niche (Uchida and Torii, 2019).

**Figure 1:**
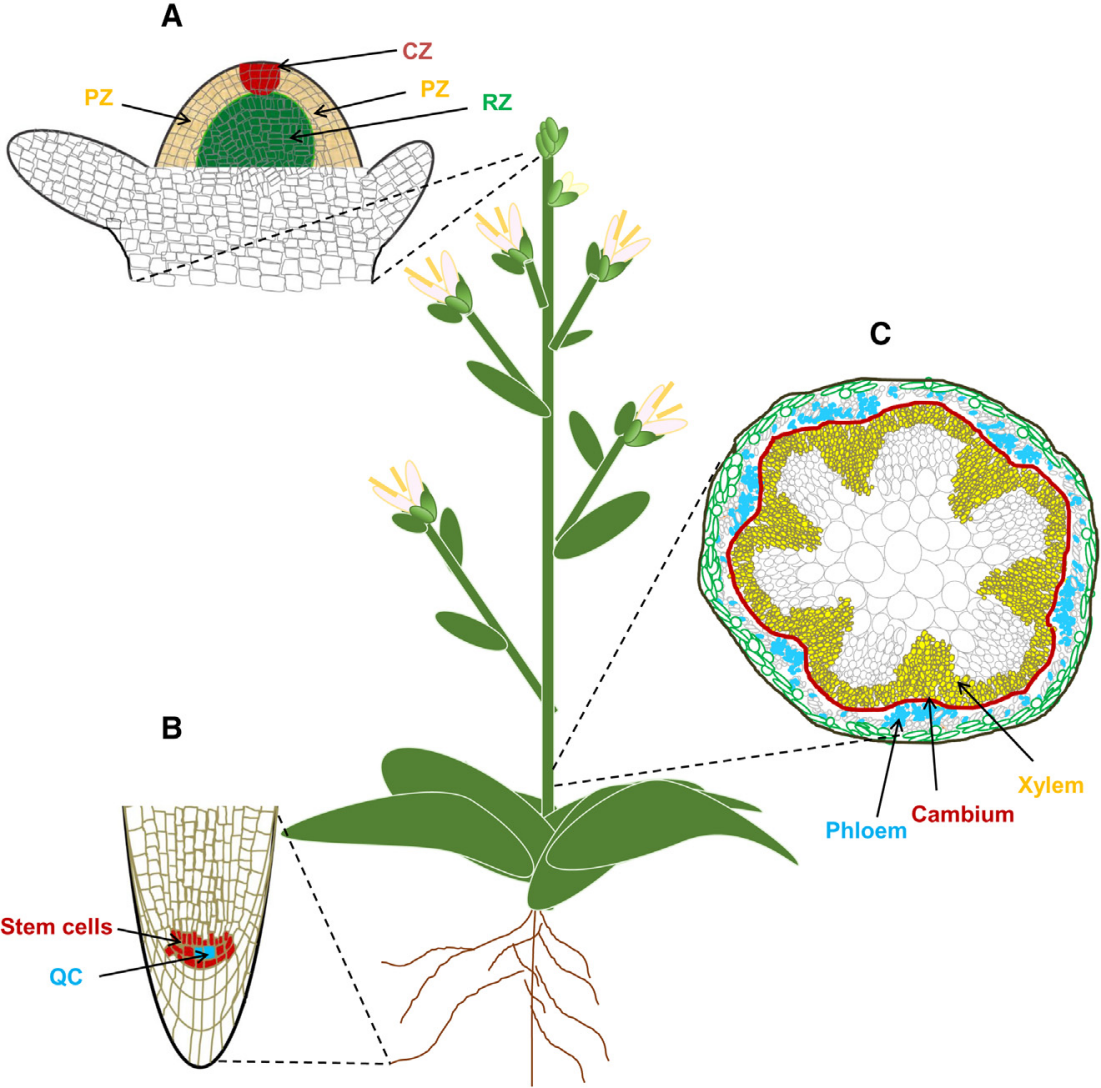
Stem cells in plants. **(A)** Schematic of a longitudinal section of the shoot apical meristem (SAM) in *Arabidopsis*. The SAM consists of three developmental zones: (i) the central zone (CZ; red) with a population of slowly dividing stem cells; (ii) the surrounding peripheral zone (yellow), where cells divide rapidly to give rise to lateral organs; and (iii) the rib zone (green), where cells differentiate into central stem tissue. **(B)** Schematic of a longitudinal section of the root apical meristem (RAM) in *Arabidopsis*. The RAM consists of a small group of cells that form the quiescent center (QC; blue) and is surrounded by stem cells (red). Signals from the QC maintain the stem cell niche of the surrounding stem cells. **(C)** Schematic of a cross-section through the *Arabidopsis* inflorescence stem. The vascular cambium meristem (VCM) is shown in red. The vascular cambium generates the xylem (yellow) and phloem (blue) by inward and outward cell division, respectively.

The SAM is a small dome-shaped structure that comprises heterogeneous cell types in three different zones (Figure 1A): (i) the central zone (CZ), with a population of slowly dividing stem cells, and the organizing center (OC) below the CZ, which controls stem cell proliferation in the CZ; (ii) the surrounding peripheral zone, where cells rapidly divide to give rise to lateral organs; and (iii) the rib zone, where cells differentiate into central stem tissue (Soyars et al., 2016). Decades of research have revealed that maintenance of stem cells in the SAM is regulated by many factors, including transcriptional regulators, receptor kinases, epigenetic marks, and hormones (Dodsworth, 2009; Somssich et al., 2016; Bustamante et al., 2016). The homeodomain transcription factor gene *WUSCHEL* (*WUS*) and *CLAVATA3* (*CLV3*), which encodes a small functional peptide, are key factors that help the SAM to remain functional and properly organized (Schoof et al., 2000; Dodsworth, 2009; Yadav et al., 2010). Regulation of stem cell activity in the SAM involves a negative feedback loop between WUS and CLV3. *WUS* is expressed in the OC, and its protein activates *CLV3* in stem cells by migrating to the CZ. In turn, the CLV3 peptide moves back to the OC, where it represses *WUS* expression (Yadav et al., 2010). This communication requires direct binding of CLV3 to the leucine-rich repeat receptor-like kinase CLV1 (Ogawa et al., 2008) and the leucine-rich repeat receptor-like protein CLV2 (Zhu et al., 2010). Recent studies have shown that FRUITFULL (FUL), a MADS-box transcription factor, has a key role in shortening *Arabidopsis* life span by repressing *WUS* expression in the SAM (Balanzà et al., 2018; Merelo et al., 2021).

Two plant hormones, cytokinin (CK) and auxin, are key players in the regulatory network that controls SAM activity (Zhao et al., 2010). CK is an essential factor for stimulating cell division in the SAM because it activates *WUS* and *CLV3* expression (Zhao et al., 2010; Chickarmane et al., 2012; Murray et al., 2012). The intercellular directionality of auxin flow at SAM zones maintained by the PIN FORMED auxin efflux carriers promotes stem cell activity and formation of lateral organs such as leaves and flowers by regulating CK signaling (Reinhardt et al., 2000; Murray et al., 2012). Enhancing the CK level in the SAM significantly prolongs the duration of fruit production in *Arabidopsis* (Bartrina et al., 2011; Niemann et al., 2015) and oilseed rape (Schwarz et al., 2020). By contrast, auxin export from the fruits to the SAM shortens the duration of fruit production (Ware et al., 2020).

It has recently been reported that the age-dependent cellular accumulation of reactive oxygen species at the *Arabidopsis* SAM shuts down stem cell activity by initiating programmed cell death (Wang et al., 2020a, 2022). Future studies are required to understand age-dependent programmed cell death in stem cells and how it contributes to the control of plant longevity.

### Role of the RAM

Plant longevity does not depend only on the activity of aboveground stem cells. Belowground, the RAM also plays a crucial role in determining lifespan (Munné-Bosch, 2014) by ensuring growth of the main root and, after branching, the lateral roots. The RAM encompasses the quiescent center (QC) and surrounding stem cells (Figure 1B; Weigel and Jürgens, 2002). The QC, as an organizer, is responsible for maintaining the surrounding stem cells (Scheres, 2007). The activity of stem cells and the maintenance of RAM proliferation activity are regulated by complex gene regulatory networks (Dubrovsky and Vissenberg, 2021; Pardal and Heidstra, 2021; Strotmann and Stahl, 2021) and hormones (Yamoune et al., 2021). QC cells have been proposed to contribute to the longer life of perennial plants (Heyman et al., 2014).

Root stem cells and their daughter cells usually die in the presence of prolonged environmental stress, whereas QC cells are highly tolerant of stress. Upon return to a non-stress condition, QC cells accelerate their cell cycle, enabling them to replace damaged stem cells. Thus, the QC likely serves as a stress-triggered reservoir of cells to ensure the replenishment of damaged stem cells (Heyman et al., 2014). Several molecular factors, including hormones (auxin, CK, ethylene, jasmonate, abscisic acid (ABA), and salicylic acid), reactive oxygen species, and transcription factors like ETHYLENE RESPONSE FACTOR 115, BRASSINOSTEROIDS AT VASCULAR AND ORGANIZING CENTER, and SCARECROW assist with activating the cell cycle machinery of QC cells in response to DNA damage-mediated death of root stem cells under environmental stress (Ubogoeva et al., 2021). ETHYLENE RESPONSE FACTOR 115 acts as a master positive regulator of the regenerative process in the stem cell niche (Heyman et al., 2013, 2016; Canher et al., 2020).

### Role of the VCM

In vascular plants, in particular woody species, cells in the stem interfascicular parenchyma differentiate into the cylindrical VCM after formation of the primary vasculature (Figure 1C). Stem cells in the VCM divide in two directions, i.e., radially inward toward the xylem and outward toward the phloem (Figure 1C; Chiang and Greb, 2019). The VCM is an important meristem whose activity gives rise to secondary growth, providing mechanical support and facilitating transport of water and nutrients throughout the plant body.

In recent years, research on *Arabidopsis thaliana* and *Populus trichocarpa*, a model tree, has demonstrated that the maintenance, proliferation, and differentiation of cambium cells require coordination of multiple signals, including hormones, regulatory peptides, and transcription factors (Immanen et al., 2016; Oles et al., 2017; Fischer et al., 2019). However, compared with SAM and RAM function, much less is known about the molecular mechanisms that govern VCM function. The TRACHEARY ELEMENT DIFFERENTIATION INHIBITORY FACTOR (TDIF)–PHLOEM INTERCALATED WITH XYLEM (PXY)–WUS-related homeobox (WOX) signaling pathway is the best-understood pathway controlling cambium activity. The peptide ligand CLE41/44/TDIF is synthesized in the phloem, from where it moves through the apoplastic space to cambium cells and binds to its cognate receptor PXY*.* The TDIF–PXY module plays an important role in maintenance and proliferation of cambium cells by activating expression of the cambium-specific *WOX4* and *WOX14* genes (Etchells and Turner, 2010; Hirakawa et al., 2010; Wang et al., 2019a). The plant hormones auxin, CK, and ethylene are major positive regulators of cambial cell proliferation (Bhalerao et al., 2016; Immanen et al., 2016; Wang et al., 2021).

Woody plants can survive for many years. Unlike the SAM, whose function is often impaired by unfavorable environmental disturbances, the VCM remains viable throughout the lifespan of a woody plant; this capacity ensures increased stem girth and the annual renewal of vascular tissue (Bowman et al., 2013). Continuous activity of the VCM has been suggested as a key factor that may inhibit whole-plant senescence in woody plants (Munné-Bosch, 2007). Protecting the genomic integrity of cambial stem cells from spontaneous mutations is probably a mechanism that underlies the growth and survival of perennial plants over many years (Burian et al., 2016). A recent study has shown that, despite considerable physiological changes and a reduction in meristem activity of the VCM during the aging of ginkgo trees (*Ginkgo biloba*), old plants continue to perform secondary growth without manifesting senescence-associated changes at the organism level (Wang et al., 2020b). This observation underscores the capacity of the VCM for controlling whole-plant senescence in trees.

## The impact of developmental phase transition on plant longevity

Post-embryonic shoot development in flowering plants is characterized by successive developmental phase transitions, beginning with the juvenile vegetative phase and followed by the adult vegetative and reproductive phases. Developmental phase transitions are the most striking examples of plant developmental changes that have a significant impact on plant longevity (Demura and Ye, 2010).

### The role of timing of the juvenile-to-adult transition

After seeds have germinated, plant development enters the juvenile phase (Poethig, 2013; Hyun et al., 2017), whose duration varies greatly among species. Fast-growing annual plants typically have a very short juvenile phase, and almost all of their shoot meristems reach the reproductive phase at the same time. By contrast, perennial plants have a long juvenile period that may take months or years, during which the transition to the adult phase is firmly prevented (Poethig, 2013). In many perennial plants, the meristems in the lower/middle parts of the plant remain in the juvenile phase while meristems in the other shoot parts are committed to the adult phase; this ability to maintain juvenility contributes to the polycarpic growth habit (Bergonzi and Albani, 2011; Muñoz-Fambuena et al., 2019).

**Figure 2:**
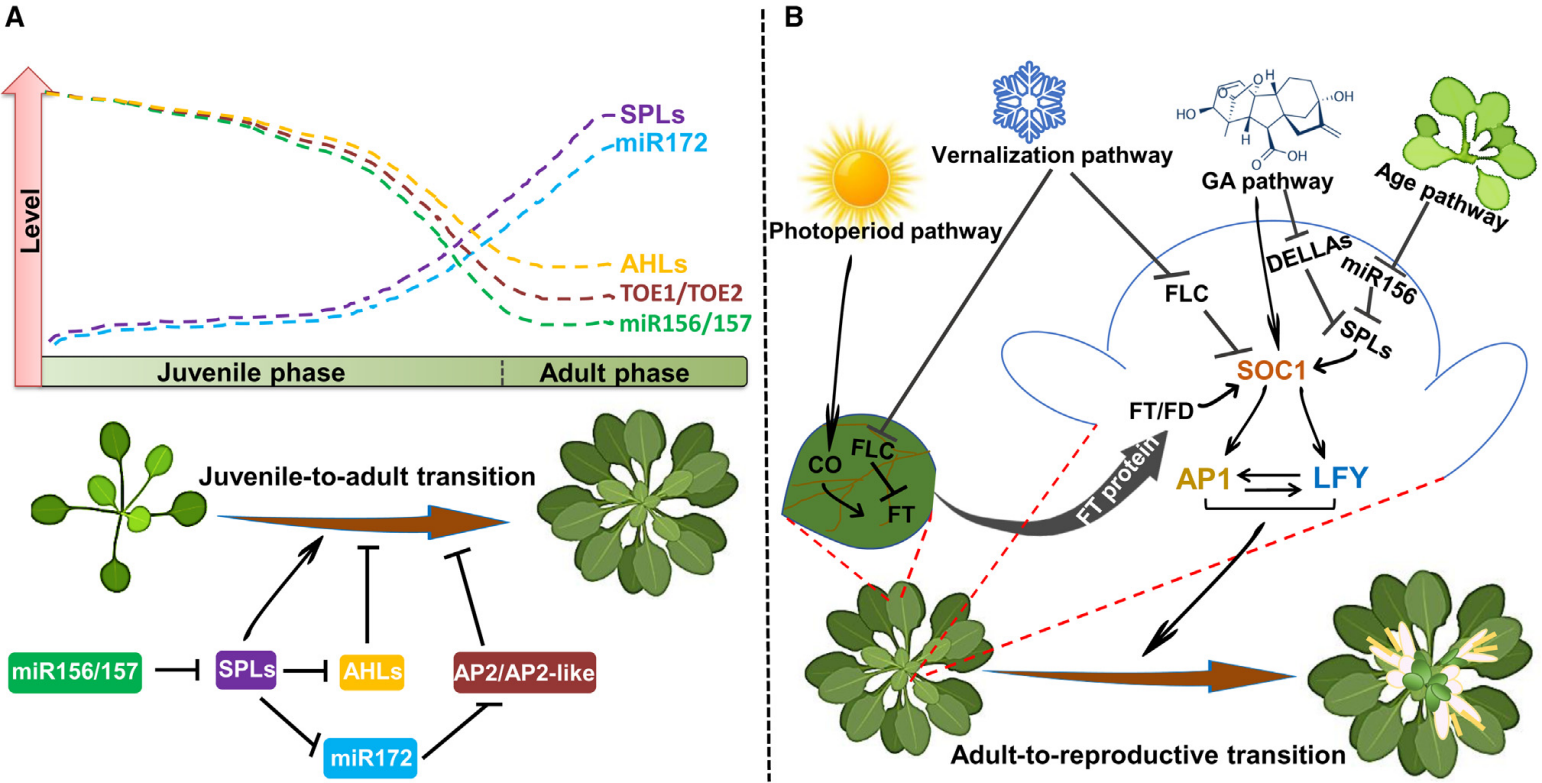
Molecular regulation of developmental phase transitions in *Arabidopsis*. **(A)** The juvenile-to-adult phase transition is regulated by *miR156*/*157* (master regulator) and AHLs through repression of *SPL* gene expression. The age-related downregulation of *miR156*/*157* and *AHL*s leads to enhanced production of SPL proteins, which promotes adult leaf morphology. The level of *miR172* increases markedly through the activity of SPLs. Increased levels of *miR172* suppress production of the TOE1 and TOE2 transcription factors, thereby allowing development of trichomes on the abaxial side of leaves. SPL abundance promotes the juvenile-to-adult phase transition in part by downregulating *AHL*s. Arrows and blunted lines indicate positive and negative regulation of the target activity, respectively. **(B)** A simplified model of the photoperiod-, vernalization-, age-, and gibberellic acid (GA)-dependent pathways of flowering regulation in *Arabidopsis*. In the photoperiod pathway, light signaling leads to CO accumulation in leaves, where CO directly activates expression of the *FT* gene. FT is a florigen protein that moves through the phloem to the SAM and triggers flowering by interacting with FD and activating *SOC1* and, as a result, *AP1* and *LFY*. In the vernalization pathway, flowering time is determined by long-term cold that leads to epigenetic silencing of the *FLC* gene. The MADS-box protein FLC represses flowering mainly by downregulating flowering-time integrators, including *FT*, *FD*, and *SOC1*. In the age-dependent pathway, a gradual decline in *miR156*/*157* level with plant age allows SPL abundance to increase, thereby activating *SOC1* and other floral integrators (not shown). Finally, GA signaling is an independent pathway that regulates flowering by activation of the *SOC1* and *SPL* genes. Subsequently, all four pathways help with transcriptional regulation of the floral integrators *FT* and *SOC1*, which promote *AP1* and *LFY* expression. *AP1* and *LFY* are necessary to complete the floral transition. Arrows and blunted lines indicate positive and negative regulation of the target activity, respectively. Created with BioRender.

Genetic studies have demonstrated that *microRNAs 156*/*157* (*miR156*/*157*) and their target transcription factors of the *SQUAMOSA PROMOTER BINDING PROTEIN-LIKE* (*SPL*) family play a central role in regulating the juvenile-to-adult transition in annual and perennial species (Wu and Poethig, 2006; Wang et al., 2011; Bergonzi et al., 2013; Zhou et al., 2013; He et al., 2018). During the juvenile phase, the gradual reduction in *miR156* expression results in increased expression of the *SPL miR156* target genes. *SPL* genes, in turn, promote progression to the adult developmental program at the SAM, resulting in transition from juvenile to adult leaf production (Figure 2A; Wu and Poethig, 2006; Xie et al., 2006; Wu et al., 2009).

SPL9 has been shown to promote some adult leaf traits by activating *miR172* transcription (Wu et al., 2009). Thus, *miR156*/*157* act upstream of *miR172* to promote adult leaf identity (Figure 2A). *miR172* promotes adult leaf identity by directly repressing members of the *APETALA2-like* (*AP2-like*) gene family, including *AP2*, *TARGET OF EAT1* (*TOE1*), *TOE2*, *TOE3*, *SCHLAFMUTZE*, and *SCHNARCHZAPFEN* (Figure 2A; Wu et al., 2009; Wang et al., 2019a, 2019b; Xu et al., 2019). However, SPLs promote other adult leaf traits, such as leaf elongation and leaf serration, independently of *miR172*.

Recently, *AT-HOOK MOTIF NUCLEAR LOCALIZED 15* (*AHL15*) and other members of the *AHL* gene family, *AHL19/20*, were shown to negatively control the juvenile-to-adult transition in *Arabidopsis* (Rahimi et al., 2022a). Genetic interaction studies indicated that SPLs promote the juvenile-to-adult transition in part by repressing *AHLs* (Figure 2A; Rahimi et al., 2022a). Further studies need to be performed to reveal the exact contribution of each AHL to control of the juvenile-to-adult transition.

### The role of timing of vegetative-to-reproductive phase transition

As development proceeds from the juvenile to the adult vegetative phase, plants acquire the competence to flower (Hyun et al., 2017). The duration of the adult vegetative phase differs among species; most annuals rapidly switch to the reproductive phase after the transition to the adult vegetative phase, but perennial plants usually remain in the adult vegetative phase for a considerable time before flowering, which allows them to generate numerous axillary meristems and produce more biomass before reproduction (Poethig, 2010).

Physiological and genetic studies have revealed that the vegetative-to-reproductive phase transition is regulated by multiple integrated endogenous and environmental cues, such as photoperiod, vernalization, age, and GA biosynthesis and signaling (Figure 2B; Amasino and Michaels, 2010; Turnbull, 2011; Andrés and Coupland, 2012; Song et al., 2013; Abe et al., 2019; Quiroz et al., 2021). Many genes, about 300, have been discovered to act in complex gene regulatory networks (GRNs) to coordinate responses to all of these cues (Blümel et al., 2015; Mourik et al., 2015; Bouché et al., 2016). In these GRNs, a few transcription factors, including *FLOWERING LOCUS C* (*FLC*), *SUPPRESSOR OF CONSTANS1* (*SOC1*), *FLOWERING LOCUS T* (*FT*), the *SPL*s, and *CONSTANS* (*CO*), act as integrators of floral pathways, affecting flowering time by integrating various environmental cues with endogenous signal pathways (Amasino, 2009; Amasino and Michaels, 2010; Andrés and Coupland, 2012; Hwang et al., 2019).

The change in photoperiod is a key seasonal cue that triggers the switch from the vegetative to the reproductive phase. The B-box-type zinc-finger transcription factor CO acts as the core component of photoperiod detection; its level increases because of light signaling in the leaf (Figure 2B; Putterill et al., 1995). Recent genetic studies have uncovered molecular mechanisms that contribute to the accumulation of CO protein (Quiroz et al., 2021). CO promotes flowering by directly activating expression of *FT*, a member of the phosphatidylethanolamine-binding protein family, and FT protein is then transported from the leaf through the phloem to the SAM (Kardailsky et al., 1999). When FT reaches the SAM, it interacts with the bZIP transcription factor FD (Taoka et al., 2011). The FT–FD complex activates transcription of several flowering-promoting genes, including *SOC1* (Figure 2B), thereby changing meristem fate from vegetative to reproductive (Samach et al., 2000; Yoo et al., 2005). Two floral meristem identity genes, *APETALA1* (*AP1*) and *LEAFY* (*LFY*), which eventually convert the vegetative SAM to an inflorescence meristem (Figure 2), act downstream of the central floral integrator SOC1 (Figure 2B; Andrés and Coupland, 2012; Matsoukas et al., 2012; Blümel et al., 2015).

Many plants have evolved a mechanism to acquire reproductive activity under prolonged periods of low temperature through a process known as vernalization. In the vernalization pathway, the MADS-box transcription factor FLC plays a central role in mediating the effect of low temperature (Figure 2B; Michaels and Amasino, 1999; Sheldon et al., 1999; Amasino, 2010). Prolonged cold exposure leads to stable repression of *FLC* by local chromatin modification (Berry and Dean, 2015). Over the past decades, extensive studies have revealed elaborate transcriptional and post-transcriptional mechanisms that regulate *FLC* in response to seasonal cues (Kim and Sung, 2014; Whittaker and Dean, 2017; Costa and Dean, 2019). FLC represses flowering mainly by downregulating *FT* and *SPL* genes in leaves and *FD* and *SOC1* in the SAM (Figure 2B; Deng et al., 2011; Matsoukas et al., 2012). Thus, FLC represses flowering pathways in the leaf and meristem. The GRNs downstream of FLC and their effects on flowering have recently been reviewed (Madrid et al., 2021).

Surprisingly, the aboveground vegetative meristems of many perennial herbs that grow in temperate climates transition to the reproductive phase in autumn before prolonged periods of low temperature in the winter (Schnablová et al., 2021). Understanding how these perennial herbs acquire the competence to flower in autumn requires further research.

Among plant hormones, GA plays a dominant role in the transition from the vegetative to the reproductive phase, particularly under non-inductive short-day conditions (Yu et al., 2012; Davière and Achard, 2013). GA signaling-induced flowering is mainly mediated by degradation of a small set of nuclear proteins, named DELLAs, which belong to the GIBBERELIC ACID INSENSITIVE REPRESSOR OF ga1-3 SCARECROW family of plant-specific nuclear proteins (Griffiths et al., 2006). In this signal transduction pathway, binding of GA to its receptor, GIBBERELLIN INSENSITIVE DWARF1, leads to interaction of GIBBERELLIN INSENSITIVE DWARF1 with DELLA proteins to trigger their proteolytic degradation (Silverstone et al., 1997; Griffiths et al., 2006; Sun, 2010; Galvão et al., 2012). GA signaling promotes the vegetative-to-reproductive phase transition mainly by activating expression of *SOC1* and *SPL* genes (Figure 2B; Fornara and Coupland, 2009; Jung et al., 2011, 2012). For a detailed description of how GA signaling contributes to flowering promotion, see Conti (2017).

Besides the critical regulatory roles of *miR156*/*157* and *miR172* in the juvenile-to-adult transition, these miRNAs also play a prominent role in the transition from the vegetative to the reproductive phase, known as the plant age-floral pathway (Poethig, 2013)*.* Molecular genetic studies have shown that the aging pathway is highly integrated into other flowering-time pathways (Wang, 2014).

## The effect of axillary meristems on plant longevity

During postembryonic development, the SAM repeatedly generates morphogenetic units called phytomers. A phytomer consists of an internode, a node with a leaf, and an axillary meristem (AM) located in the leaf axil (Grbić and Bleecker A, 2000; Wang et al., 2018). AMs are newly formed meristems harboring stem cells that have the same developmental identity as the SAM. Recent genetic and physiological studies have uncovered several genes, phytohormones, and regulatory pathways that control AM formation (Balkunde et al., 2017; Scofield et al., 2018; Zhu and Wagner, 2020).

The AMs, when formed, develop into axillary buds comprising a meristem and a few leaf primordia; these buds may enter growth immediately or may initially be inhibited, developing into dormant axillary buds. The dormant buds remain inhibited as long as they are triggered by internal factors or environmental cues; later they grow out, leading to formation of lateral branches. The outgrowth of dormant axillary buds and the developmental fate of their meristems is the main determinant of plant architecture and longevity (Pautler et al., 2013).

### The role of AM developmental phase identity for longevity of annual plants

**Figure 3:**
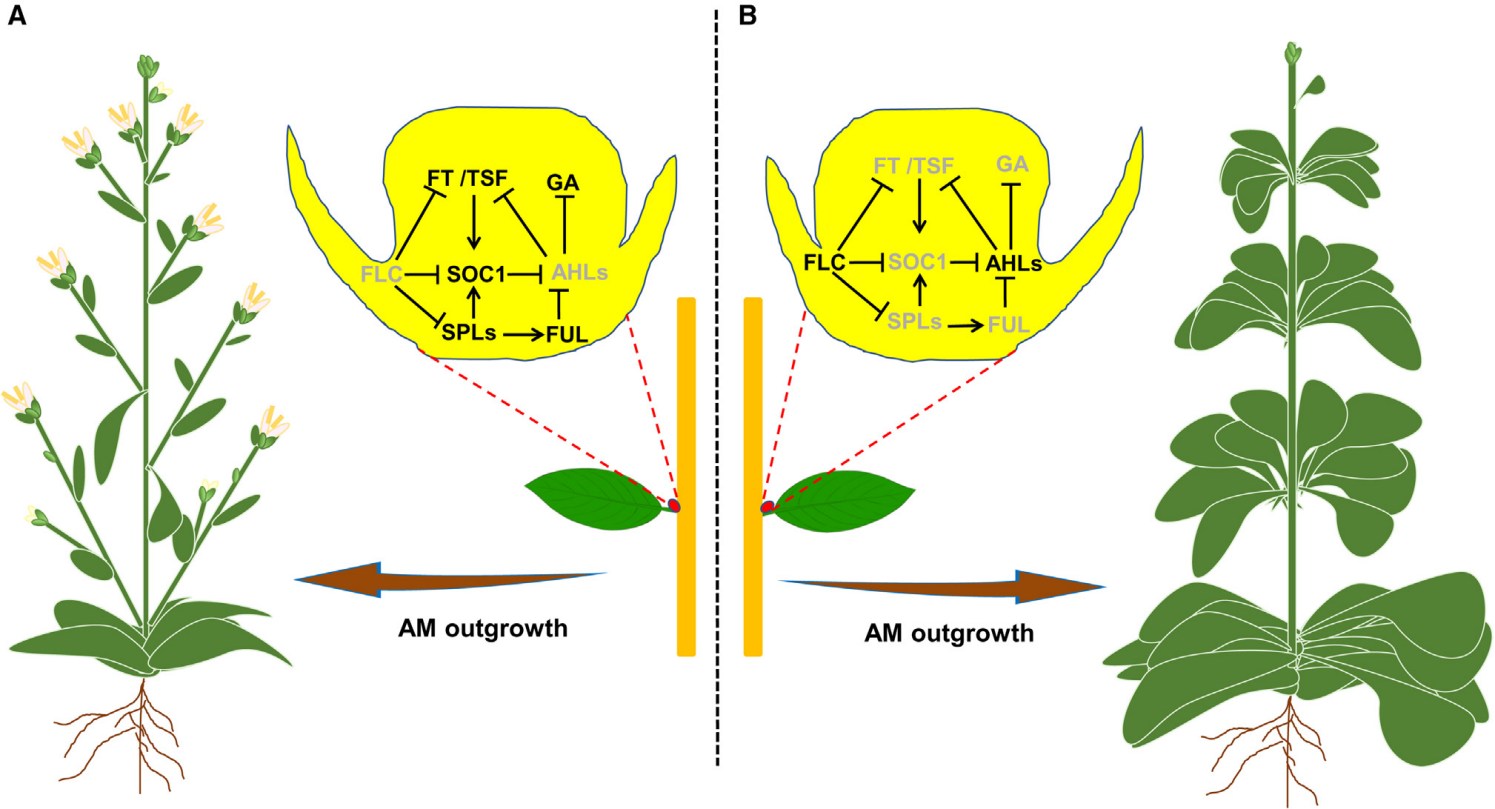
Developmental phase identity of AMs. **(A and B)** Schematic of the GRN that mediates the immediate development of AMs into inflorescences in *Arabidopsis* accession Col-0 **(A)** or extends vegetative identity to result in an increased number of rosette leaves on basal nodes and aerial rosettes in accession Sy-0 **(B)**. Blunt-ended lines indicate repression, and arrows indicate promotion. Black indicates genes that are expressed; gray represents repressed genes.

In annual plants, AMs can adapt to different developmental phase identities, and this is a major determinant of plant longevity and shoot branching pattern. In *A. thaliana* and many other annual flowering plants, the vegetative identity of AMs is strongly suppressed, and the majority of AMs immediately initiate reproduction and develop into inflorescences (Figure 3; Amasino, 2009; Davies and Gan, 2012); thus, energy is largely funneled toward reproductive activities, eventually leading to death of the plant body (Thomas, 2013). This growth feature gives rise to a simplified morphology and limited branch numbers and biomass. However, in some *Arabidopsis* accessions such as Sy-0, the immediate reproductive identity of the AMs is prevented by maintaining the vegetative identity (Poduska et al., 2003). This change in the pattern of AM developmental phase identity in Sy-0 gives rise to an enlarged basal rosette and aerial rosette formation in the axils of cauline leaves (Figure 3), significantly extending longevity and the period during which the plant can produce seeds (Poduska et al., 2003). Over time, the vegetative AMs of Sy-0 can grow out into inflorescences, leading to higher branching. Despite the important role of AM developmental phase identity in shoot architecture and crop productivity, the molecular mechanisms that suppress the reproductive identity of AMs are largely unknown.

The key factors that control vegetative-to-reproductive phase transition act as important determinants of plant longevity by regulating the vegetative activity of AMs during the reproductive phase. As observed in Sy-0, a significant extension of longevity has been reported in gain- and loss-of-function mutants of key flowering repressors or activators, respectively, in *A. thaliana*. For example, a double mutant of the flowering-time genes *SOC1* and *FUL* produces many aerial rosettes, leading to an increased lifespan (Melzer et al., 2008). Recently, enhanced expression of *AHL15* and several *AHL15* homologs was shown to lead to maintenance of vegetative identity in *Arabidopsis* AMs (Karami et al., 2020). *AHL15* acts directly downstream of SOC1 and FUL as a central floral repressor of AMs, and AHL15 suppresses the reproductive identity of AMs in part by reducing GA biosynthesis (Karami et al., 2020).

SPL proteins also promote the reproductive identity of AMs (Rahimi et al., 2022a). Similar to overexpression of *AHL15*, repression of *SPL* by *miR156* overexpression maintains AMs in the vegetative phase during flowering, resulting in increased lifespan and extension of the seed production period, and similar results were observed in the *spl9 spl15 double mutant* (Rahimi et al., 2022a). In support of these findings, SPLs suppress the expression of *AHL* genes (Rahimi et al., 2022a) and promote *SOC1* and *FUL* expression (Wang et al., 2009a, 2009b). Thus, SPLs promote the reproductive identity of AMs by upregulating *SOC1* and *FUL*, which subsequently leads to suppression of *AHL* expression (Figure 3).

In *Arabidopsis*, FT and TSF proteins can move to the axillary bud from the subtending leaf and promote the floral transition in AMs (Niwa et al., 2013). AHL22 and AHL20 repress *FT* and *TSF* expression (Yun et al., 2012; Tayengwa et al., 2020). Therefore, suppression of the floral transition in AMs by AHL15 may be mediated in part through repression of *FT* and *TSF* expression in leaf tissues.

AHLs are nuclear proteins that have only been studied relatively recently; their mechanisms of action remain to be clarified. However, these proteins are believed to act through chromatin modification, and evidence suggests that AHLs change higher-order chromatin structure (Lim et al., 2007; Karami et al., 2021). *AHL22* has been shown to repress *FT* expression by binding to the *FT* promoter, where it may modulate the epigenetic signature around its binding region (Yun et al., 2012).

FLC acts as a repressor of vegetative-to-reproductive meristem transition through negative regulation of the *FT*, *TSF*, *SOC1*, and *SPL15* flowering-time genes in response to seasonal cues (Deng et al., 2011; Matsoukas et al., 2012). Elevated levels of the *FLC* transcript have been suggested to underlie the formation of enlarged basal and aerial rosettes and the increased lifespan of Sy-0 and several other *Arabidopsis* accessions (Poduska et al., 2003; Wang et al., 2007). However, a key role for *FLC* in maintaining the vegetative identity of AMs in Sy-0 has not yet been demonstrated. It would be interesting to explore the detailed genetic interactions between key floral regulator genes such as *FLC*, *FT*, *SOC1*, *AHL*s, and *SPLs* in controlling the longevity of longer-lived *Arabidopsis* accessions.

Based on the above-mentioned findings, we propose a model of the GRN that controls phase identity of axillary shoot meristems in *Arabidopsis* (Figure 3).

### The role of axillary bud suppression at the last stage of development in monocarpic plants

In many plant species, the AMs initially develop into dormant axillary buds, but most of them resume growth at later times. The outgrowth of dormant axillary buds is a plastic developmental process regulated by a wide range of endogenous factors and several environmental factors (Djennane et al., 2014; Rameau et al., 2015; Zhu and Wagner, 2020).

In monocarpic annual plants, outgrowing axillary buds are suppressed at the last stage of development, and this leads to early cessation of lateral shoot production and reduced plant longevity (Guo and Gan, 2011). In *Arabidopsis*, *MYB DOMAIN PROTEIN 2* (*MYB2*) plays a key role in suppressing axillary bud outgrowth at late developmental stages (Guo and Gan, 2011). Knockout mutation of this gene results in an extensive outgrowth of axillary buds at late stages of development and prolongs plant longevity (Guo and Gan, 2011). *MYB2* is expressed at basal internodes, and it prevents axillary bud outgrowth by repressing CK biosynthesis.

Elevated expression of *AHL15* under control of the *MYB2* promoter stimulates axillary bud outgrowth by increasing endogenous CK levels (Rahimi et al., 2022b). Interestingly, in wild-type *Arabidopsis* plants (Col-0 accession), *AHL15* expression is strongly downregulated in axillary buds at their last stage of development (Karami et al., 2020). It thus appears that suppression of axillary bud outgrowth is triggered by lowered CK levels, a mechanism mediated by activation and repression *of MYB2* and *AHL15*, respectively.

### Role of developmentally controlled AM phase identity in establishing the longevity of perennial plants

The ability to maintain functional AMs after a successful round of offspring production is an important determinant of polycarpic growth behavior (Kiefer et al., 2019). Indeed, the life history of many perennial herbaceous plants, such as *Arabis alpina* and *Arabidopsis lyrata*, relies on maintaining some AMs in the vegetative phase in a given growth season, thereby ensuring subsequent cycles of growth during the next season, while other AMs undergo floral transition, senesce, and set seeds (Amasino, 2009). Despite considerable interest in lifespan strategies, the molecular mechanisms that determine seed setting-linked death in monocarpic plants and survival of polycarpic plants after multiple rounds of flowering and seed setting are still largely unknown. Analysis of the genetic basis of polycarpic growth habits has recently begun in some Brassicaceae species.

The flowering repressor *PERPETUAL FLOWERING1* (*PEP1*; an ortholog of *Arabidopsis FLC*), which is transcriptionally regulated by vernalization, controls polycarpic growth habits in *A. alpina* (Wang et al., 2009a, 2009b). Lack of epigenetic memory at the *PEP1* locus during cold exposure leads to downregulation of *PEP1*, which allows the vegetative-to-reproductive transition of meristems during winter. By contrast, shoot meristems of the *pep1* mutant acquire competence to flower without a cold period, shortening the vegetative phase, and many AMs readily flower. Interestingly, nonfunctional *PEP1* alleles were found in *A. alpina* accessions that undergo death after the first round of offspring production (Albani et al., 2012; Hughes et al., 2019).

Although PEP1 remains stably silenced in meristems after transition to the reproductive phase during cold exposure, chromatin modifications at the *PEP1* locus are restored to their original levels when temperatures increase in the spring, leading to increasing *PEP1* expression in newly formed AMs in the axils of leaf primordia close to the SAM (Albani et al., 2012; Hughes et al., 2019). AMs formed on different axillary branches of *A. alpina* are adapted to different developmental phases, from the juvenile to the adult vegetative and reproductive phases (Park et al., 2017; Lazaro et al., 2018). Interestingly, vernalization does not lead to silencing of *PEP1* in juvenile meristems because they retain a vegetative fate during cold exposure. These juvenile meristems, together with new adult vegetative AMs formed after increases in *PEP1* during the summer, support the subsequent cycle of growth in *A. alpina* (Wang et al., 2009a, 2009b; Lazaro et al., 2018).

The vernalization-dependent pathway controlling the life history of *A. alpina* is also regulated by *miR172*. The complementary expression patterns of *miR156* and *miR172* in *Arabidopsis* are not observed in *A. alpina*; the reduction in *miR156* level is uncoupled from increases in *miR172* abundance, whereas the level of *miR172* is upregulated independently of the ageing pathway by cold temperatures (Bergonzi and Albani, 2011; Lazaro et al., 2019). Activation of *miR172* by prolonged exposure to cold during the winter leads to repression of *PEP2*, a paralog *AP2-like* transcription factor. Similar to those of the *pep1* mutant, the shoot meristems of loss-of-function *PEP2* plants do not need a cold period to become reproductive. PEP2 represses flowering in part by enhancing the expression of *PEP1* but also acts independently of PEP1 (Lazaro et al., 2019). Thus, PEP1 and PEP2 play a dual role in suppression of floral transition. The mechanism by which prolonged cold exposure promotes the expression of *miR172* in *A. alpina* is not yet known. In *Arabidopsis,* besides an increase in *miR156* level, activation of CK signaling molecules (i.e., the type B response regulators [ARRs] AAR1, ARR10, and ARR12) in the shoot results in an increase in *miR172* levels (Werner et al., 2021). Therefore, upregulation of *miR172 by* prolonged cold exposure may be mediated by activation of CK signaling.

A decline in *miR156* levels and a subsequent increase in expression of *SPL* genes are essential for induction of flowering by prolonged cold exposure in *A. alpina*; AMs that constitutively express *MIR156* maintain their vegetative fate in response to vernalization, and only after a decline in *miR156* levels are they competent to enter the reproductive phase (Bergonzi et al., 2013). Enhancing *SPL* expression by reducing *miR156* activity in *miR156* target mimic (*35S:MIM156*) lines (Franco-Zorrilla et al., 2007) increases the number of AMs that respond to vernalization, whereas prolonged cold exposure cannot induce flowering in an *SPL15* loss-of-function mutant of *A. alpina* (Hyun et al., 2019). These results indicate that the age pathway for control of polycarpic life history in *A. alpina* is independent of the vernalization pathway.

*Cardamine flexuosa,* another perennial herbaceous Brassicaceae plant, shows a different connection between the aging and vernalization pathways. The polycarpic life history of *C. flexuosa* is also mediated by prolonged cold exposure, but unlike *A. alpina*, its age-dependent decline in *miR156* level is associated with increased expression of *miR172*, independent of the vernalization pathway (Zhou et al., 2013), which causes repression of its target *TOE1* and subsequently leads to repression of *PEP1* expression (Zhou et al., 2013). These findings indicate that polycarpic behavior in Brassicaceae can be controlled by different mechanisms.

An *Arabidopsis* double mutant of the flowering genes *SOC1* and *FUL* was reported to display a polycarpy-like growth habit (Melzer et al., 2008). Interestingly, the PEP1*/*PEP2-induced polycarpic behavior of *A. alpina* is also caused by suppression of *AaSOC1* (Bergonzi et al., 2013) and *AaFUL* (Lazaro et al., 2019) expression. These studies indicate that advances in understanding the molecular mechanisms that control monocarpic life strategies can help to clarify how polycarpic plants can live for many years. Elevated levels of *AHL15* have been reported to enable *Arabidopsis* and tobacco to survive after the first seed set, which resembles the situation in polycarpic plants (Karami et al., 2020). The polycarpy-like behavior of *soc1 ful* plants contributes to the negative regulation of *AHL15*. Transcription of *AHL15* is repressed by binding of SOC1 and FUL to the *AHL15* promotor; thus, the two transcription factors SOC1 and FUL suppress vegetative identity of AMs by repressing *AHL15* (Karami et al., 2020). Although the mechanism by which AHL15 suppresses the vegetative identity of AMs is not yet known, AHL15 represses expression of *GA3OX1*, *GA20OX1*, and *GA20OX2*, which encode major enzymes of GA biosynthesis (Karami et al., 2020). Interestingly, transcription of some GA biosynthesis genes also increases during prolonged cold exposure in *A. alpina* (Tilmes et al., 2019). Therefore, *AHL* genes might act downstream of AaSOC1 and AaFUL and upstream of GA to suppress entry of AMs into the reproductive phase and induce perennial life history*.*

Several lines of evidence have shown that GA plays an important role in control of AM fate and perennial life history outside of the Brassicaceae family. A deletion in *GA20OX2*, which is highly expressed in axillary buds of woodland strawberry (*Fragaria vesca*), leads to a strong trade-off between flowering and runner production (Tenreira et al., 2017). *GA20OX2* was also found to be a major allele frequency outlier between annual and perennial ecotypes of *Mimulus guttatus* (Gould et al., 2017), and GA application to perennial *M. guttatus* plants resulted in an annual-like morphology (Lowry et al., 2019).

*TERMINAL FLOWER1* (*TFL1*), a homolog of *A. thaliana FT*, is another important gene that controls the polycarpic growth habit of perennial plants (Jensen et al., 2001; Périlleux et al., 2019). In *Arabidopsis*, TFL1 functions as a key regulator of inflorescence meristem indeterminacy and as a negative regulator of flowering time (Shannon and Meeks-Wagner, 1991; Simon et al., 1996; Ratcliffe et al., 1998). Orthologs of *TFL1* also play important roles in determining whether AMs remain vegetative or commit to flowering in perennials, as demonstrated in *Malus domestica*, *P. trichocarpa*, *F. vesca*, and *Lolium perenne* (Jensen et al., 2001; Mohamed et al., 2010; Tenreira et al., 2017).

In *Rosa rugosa*, the flowering repressor *KSN*, a homolog of *Arabidopsis TFL1*, takes over the regulatory role of FLC in response to seasonal cues. Expression of *KSN* is downregulated during vernalization, and its expression increases upon return to warmer conditions in summer, suppressing further flower formation (Iwata et al., 2012; Randoux et al., 2012; Bendahmane et al., 2013). Insertion of a transposon results in a null allele of *KSN* in *Rosa chinensis*, causing continuous flowering in the summer (Iwata et al., 2012). The mechanism by which TFL1 suppresses floral transition in AMs in perennials remains to be clarified.

### Sources of meristems to ensure growth of perennial plants in the next growing season

In perennial plants, maintaining some AMs in the vegetative phase and producing new vegetative AMs during the reproductive phase are the main means of ensuring development of new shoots in the next growing season (Amasino, 2009). These meristems are located in the leaf axils at stem nodes, and they resume growth in the spring after a dormant winter period. In some species (about 10% in Central Europe) not only AMs on stems but also adventitious meristems on hypocotyls and roots play a role in perennation (e.g., *Euphorbia esula*, *Cirsium arvense*) (Klimešová et al., 2015; Bartušková et al., 2021). Anatomical investigations revealed that meristematic cells in the root VCM are probably essential for formation of adventitious vegetative meristems (Bartušková et al., 2021; Martínková et al., 2023).

In trees, a new population of vegetative AMs is typically produced annually on new twigs as apical and subapical meristems (Costes et al., 2014), and in herbs, the axillary and adventitious meristems that ensure perennation are located on stem bases or specialized belowground organs like rhizomes, tubers, bulbs, or roots (Ott et al., 2019; Guo et al., 2021). These belowground bud-bearing organs often provide plants with the capacity for vegetative multiplication (Herben and Klimešová, 2020).

After a period of flowering, the flower or inflorescence meristems of some perennial plants revert to vegetative tissues, a mechanism that ensures subsequent cycles of growth during the next growing season (Tooke et al., 2005; Amasino, 2009; Bergonzi and Albani, 2011). Reversion of flowering is a relatively rare developmental phenomenon that occurs in response to unusual environmental conditions unsuitable for sustaining reproductive growth, such as high or low temperature, low humidity, or low light (Tooke et al., 2005).

## Protection of meristems in perennial plants

In perennials, aboveground buds (in woody plants) or underground buds formed on organs like bulbs, corms, rhizomes, stem collars, xylopodia, tuberous roots, or rhizophores (in herbaceous plants) enter a state in which the meristems stop growing and enter dormancy to ensure their survival over winter and enable growth resumption at warmer temperatures the following spring (Horvath et al., 2003). Buds generally enter dormant states through paradormancy, endodormancy, or ecodormancy (Lang et al., 1978). In paradormancy, meristem growth is suppressed by physiological factors from other plant parts. Endodormancy is mainly induced by short days and/or decreased temperatures in autumn, which enable buds to become tolerant to low winter temperatures. After entering endodormancy, buds cannot sprout, even under favorable conditions. In ecodormancy, unfavorable environmental conditions in the final stage of dormancy suppress the outgrowth of dormant buds that have acquired the ability to resume growth (Lang et al., 1978).

Bud dormancy is a complex process that is mainly driven by a combination of genetic and environmental factors (Horvath et al., 2003; Yang et al., 2021). Despite significant efforts over the past decade, the molecular mechanisms that regulate bud dormancy still remain largely unknown. However, recent molecular-genetic studies in woody perennials have revealed that endodormancy is regulated by a set of MADS-box domain transcription factors; i.e., DORMANCY-ASSOCIATED MADS-BOX 1–6 (DAM1–DAM6) (Bielenberg et al., 2004; Falavigna et al., 2019). DAM1–DAM6 are closely related to the *Arabidopsis* floral repressor SHORT VEGETATIVE PHASE and AGAMOUS-like 24, which respond to environmental factors, including temperature and photoperiod (Bielenberg et al., 2004; Falavigna et al., 2019). Decreased temperature, an important factor in dormancy induction, activates *DAM* genes through epigenetic modifications at the histone level (Leida et al., 2012; Singh et al., 2018) and through DNA methylation (Rothkegel et al., 2017).

Plant hormones have been found that play a critical role in regulating bud dormancy (Liu and Sherif, 2019). In particular, ABA is known as a central regulator of bud dormancy (Liu et al., 2021). Recent studies have shown that an increasing level of endogenous ABA induces bud endodormancy through callose deposition, plasmodesmata closure (Tylewicz et al., 2018), and activation of *DAM* genes (Wu et al., 2017). However, the underlying molecular processes are still largely unknown.

## Concluding remarks and perspectives

The role of stem cells in determining longevity variation is a common theme in angiosperms. In this review, we discussed how the activity or fate identity of stem cells determines plant longevity variation and which internal factors and environmental signals affect them.

However, only few genes involved in regulating plant longevity have been identified to date, and we lack a good understanding of the molecular mechanisms by which the activity and fate of stem cells contribute to plant longevity variation. Understanding the molecular mechanisms that control the phase identity of stem cells will be important for understanding plant longevity variation. Therefore, future research is necessary to elucidate in detail the genetic and molecular mechanisms that control stem cell phase identity in the context of plant longevity variation.

Environmental stresses such as drought, salinity, flooding, chilling, wounding, heavy metal content, and UV light irradiation can induce the death of stem cells in apical and other meristems. Therefore, in addition to stem cell activity or fate identity, maintenance of stem cell proliferation or recovery of stem cells to survive environmental stress plays an important role in determining plant longevity. Identifying components and network-based regulatory mechanisms by which the stem cell niche responds to environmental stress is becoming essential for deciphering plant environmental adaptation.

Perennial grain plants have great potential for reducing global greenhouse gas emissions and providing economic benefits associated with their cultivation. Therefore, domesticating new perennial grain crop species and converting annual grain plants into perennial plants have been considered promising approaches for ensuring food security in future environments (Werling et al., 2014; Friedman, 2020; Lundgren and Des Marais, 2020). Despite interest in accelerating the development of sustainable agriculture based on perennial grain crops, few efforts have been made to integrate perennial crops into agricultural contexts. Research on the regulatory mechanisms behind perenniality not only provides a better understanding of the mechanisms and components of perenniality but also offers opportunities for developing sustainable agriculture.

In the near future, efficient breeding strategies based on integrated information from vast archives of genetic materials, complete genome sequences, transcriptome data, and functionally characterized genes, as well as CRISPR-based genome editing technologies and efficient crop transformation methods, will accelerate the improvement of crop longevity and may enable the conversion of annual into perennial grain plants.

## Funding

This study was supported by 10.13039/501100001717Leiden University (Leiden Institute of Physics and Institute of Biology Leiden).

## Author contributions

A.R. and O.K. drafted the manuscript. B.M.-R. assisted with its revision. All authors agreed with the submission of the final manuscript.
